# Effects of Different Thermo-Hygrometric Conditions on Ecological Interactions Between the Warehouse Pirate Bug, *Xylocoris flavipes* (Hemiptera: Anthocoridae), and Its Prey, *Liposcelis decolor* (Psocodea: Liposcelididae)

**DOI:** 10.3390/insects16090888

**Published:** 2025-08-25

**Authors:** Augustine Bosomtwe, George Opit, Brad Kard, Kristopher Giles, Carla Goad

**Affiliations:** 1 Department of Entomology and Plant Pathology, Oklahoma State University, 127 Noble Research Center, Stillwater, OK 74078, USA; george.opit@okstate.edu (G.O.); b.kard@okstate.edu (B.K.); kris.giles@okstate.edu (K.G.); 2 CSIR-Plant Genetic Resources Research Institute, Bunso P.O. Box 7, Ghana; 3 Department of Statistics, Oklahoma State University, 301 Mathematics, Statistics and Computer Sciences, Stillwater, OK 74078, USA; carla.goad@okstate.edu

**Keywords:** psocid, stored-product pest management, biological control, anthocoridae, warehouse pirate bug

## Abstract

Physical conditions in grain storage environments influence trophic interactions between predators and their prey and can affect the effectiveness of biocontrol agents. The study aimed to assess the potential of *Xylocoris flavipes* (Reuter) (Hemiptera: Anthocoridae), to manage *Liposcelis decolor* (Pearman) (Psocodea: Liposcelididae). *Liposcelis decolor* population suppression and *X*. *flavipes* progeny production were assessed at five predator–prey (P-P) ratios (0:240, 1:240, 2:240, 3:240, and 5:240), four temperatures (20, 24, 28, and 32 °C), and three relative humidities (RH) (63, 75, and 85%) over 40 days at 0:24 (L:D) photoperiod in the laboratory. Compared with the Control P-P ratio of 0:240 (no predators), prey suppression >97% was achieved across all predator release ratios. At 32 °C and 75% RH, which are the optimal conditions for *L. decolor*, 3985.13 ± 255.45 prey survived in the Control P-P ratio compared with 19.85 ± 2.47–115.73 ± 8.99 found for the four P-P ratios with the predator, representing prey reduction of 97.10–99.50%. Temperature influenced *X. flavipes* progeny production, which was greatest at 28 °C and a P-P ratio of 1:240. Suppression caused by *X*. *flavipes* demonstrates its potential as a biological control agent to manage psocid infestations in stored commodities.

## Simple Summary

Stored-grain psocids have natural tolerance to insecticides including phosphine; thus, managing them with conventional methods is difficult. *Xylocoris flavipes*, commonly known as the warehouse pirate bug, is a predator of stored-product insect pests with potential for use as a biocontrol agent to manage psocids. This study demonstrates the potential of *X*. *flavipes* to manage *Liposcelis decolor*, a psocid species with high tolerance to phosphine. Prey suppression and progeny produced by *X. flavipes* were assessed at five predator–prey (P-P) ratios (0:240, 1:240, 2:240, 3:240, and 5:240), four temperatures (20, 24, 28, and 32 °C), and three relative humidities (RH) (63, 75, and 85%) over 40 days at 0:24 (L:D) photoperiod in the laboratory. The study found that *X. flavipes* preyed on *L*. *decolor* and caused ~97.17–99.46% *L*. *decolor* population suppression for 1:240, 2:240, 3:240, and 5:240 P-P ratios under the different laboratory temperatures and RH. *Xylocoris flavipes* also increased its progeny and established itself at both low and high release ratios across the tested temperatures and RH conditions. Suppression caused by *X*. *flavipes* demonstrates its potential as a biological control agent to manage psocid infestations in stored commodities.

## 1. Introduction

Psocids (Psocodea: Liposcelididae) are stored-product insect pests of economic importance because of their tolerance and resistance to insecticides, their ability to cause significant weight losses of stored grains by the consumption of germ and endosperm, and the risk they pose in trade due to the potential rejection of food commodities infested with psocids [1,2,3]. Psocids are highly prolific, and controlling them with phosphine—the most commonly applied and usually effective insecticide against lepidopteran and coleopteran pests is challenging [4,5]. Many species of natural enemies occur in the storage environment and represent potential biological control agents against stored-product insect pests including psocids [2,6,7,8]. Natural enemies, including *Xylocoris flavipes* (Reuter) (Hemiptera: Anthocoridae), *Cheyletus eruditus* (Schrank) (Trombidiformes: Cheyletidae), *Cheyletus malaccensis* Oudemans, *Trichogramma evanescens* Westwood (Hymenoptera: Trichogrammatidae), and *Habrobracon hebetor* (Say) (Hymenoptera: Braconidae), are known to be associated with stored-product insect pests in tropical and temperate regions [4,6,9,10,11]. One of the most common natural enemies that preys voraciously on eggs, larvae, and pupae of stored-product beetles and moths is *X. flavipes*, commonly known as the warehouse pirate bug [9,12,13]. *Xylocoris flavipes* is a generalist predator that is widely distributed in storage and processing facilities and has the natural ability to penetrate grain mass [6,11]. The predator has a high capacity to increase in population relative to its prey and destroys large numbers of prey when abundant [8,9]. Recent studies [8,14] have demonstrated the potential of *X*. *flavipes* to manage all mobile stages of *Liposcelis decolor*, a psocid species with considerable tolerance to phosphine [5]. Laboratory studies found that phosphine discriminating doses for *L. decolor* were 249.76 and 194.5 ppm over 20 h and 72 h of fumigation, respectively [5]. These concentrations of phosphine are quite high.

Biotic and abiotic factors influence trophic-level interactions between predators and their prey and can significantly affect the effectiveness of biocontrol agents in pest management [15,16]. Two key abiotic factors that regulate arthropod population and can influence the overall outcome of predator–prey ecological interactions are temperature and relative humidity [17,18]. Temperature and relative humidity regulate arthropod population dynamics by influencing the rate of reproduction, development, and death [17]. Predators and prey may require different conditions for growth, development, and survival. For instance, the optimal temperature for growth and reproduction of *X*. *flavipes* ranges from 28 to 31 °C at 63–70% RH. However, its life cycle can be completed at temperatures above 35 °C [19,20,21]. Depending on the psocid species, development from egg to adult can generally occur at temperatures between 20 and 42.5 °C, with optimal temperature and relative humidity for growth ranging between 32.5 and 35 °C and 70–80% RH [22,23,24]. In storage ecosystems, predator–prey interactions are further complicated by certain biotic factors including competition, cannibalism, interference, intraguild predation due to the existence of natural enemy complex or conspecifics [15,16]. These factors may critically influence predator establishment and impact long-term success of biocontrol programs. Again, predation, reproduction, and establishment of predators that enable them to suppress populations of pests can be significantly influenced by prey-related factors including prey nutrition and type, and their spatial and seasonal abundance [25,26].

Successful implementation of biological control programs requires careful consideration of predator release ratios at a given pest density that would be sufficient to achieve maximum pest suppression [27]. Optimal release ratios depend on multiple factors including predator foraging capacity, environmental conditions, and spatial distribution of both predator and prey within the storage environment. Previous studies on the functional and numerical responses of *X*. *flavipes* have demonstrated that the predator can consume and reproduce on both nymphs and adults of *L. decolor* at a laboratory temperature of 28 ± 1 °C and 63 ± 5% RH [8,14]. However, there is limited information on how release ratios and physical conditions in storage environments affect the effectiveness of *X*. *flavipes* for managing psocid populations. Understanding predator–prey interactions within the broader ecological conditions of storage environments is essential for successful biocontrol. Because several abiotic and biotic factors can affect the performance of biocontrol agents, an evaluation of predator-prey interactions based on variables including predator–prey ratio, temperature, and RH conditions similar to those found in commodity storage environments would be useful for efficient deployment of biocontrol agents. Therefore, the objective of the current study was to assess the ecological interactions between the predatory warehouse pirate bug, *X*. *flavipes,* and its prey, *L*. *decolor*, under different P-P ratios, temperatures, and RH. Prey suppression and number of progeny produced by *X*. *flavipes* were estimated under the different P-P ratios and thermo-hygrometric regimes. This study provides baseline information necessary for further field evaluation of *X*. *flavipes* to enable its incorporation into current IPM programs for controlling psocids.

## 2. Materials and Methods

### 2.1. Rearing of Liposcelis decolor

Cultures of *L*. *decolor* were maintained in the laboratory as described in [7,28], and used as prey in this study. Only adult females of *L. decolor* (hereafter referred to as adult♀ *L. decolor*) that were selected from the laboratory cultures were used for this study.

### 2.2. Rearing of Xylocoris flavipes

Initial cultures of *X. flavipes* were acquired from Biologische Beratung GmbH, Berlin, Germany. Subsequent cultures of *X. flavipes* were maintained on *L. decolor*, as described in [8,14]. About 50 pairs of *X. flavipes* males and females were initially introduced into the jars with abundant supply of *L. decolor* as prey, as described in [8,14]. The jars containing both *X. flavipes* and *L. decolor* were placed in plastic boxes with 63 ± 2% RH. The boxes were subsequently placed inside a growth chamber and maintained at 28 ± 1 °C and a 0:24 (L:D) photoperiod for the *X. flavipes* to multiply and establish as described in [8,14]. The laboratory rearing conditions ensure standardization and consistent baseline assessment of *X*. *flavipes* biocontrol potential under optimal conditions. Adult females of *X. flavipes* (hereafter referred to as adult♀ *X. flavipes*) were selected and used for this study.

### 2.3. Experimental Arenas

A 5.0-cm diameter basal Petri dish with a 5.5-cm diameter lid constituted an experimental arena as described in [8,14]. Arenas were prepared as described in [7,28]. Adult♀ *L. decolor* were provisioned with 5.0 g of cracked wheat in each basal Petri dish.

### 2.4. Predation and Progeny Production of Xylocoris flavipes

*Liposcelis decolor* population suppression levels by *X*. *flavipes* were assessed at different P-P ratios, temperatures, and RH over a 40-day study. Five P-P ratios (0:240, 1:240, 2:240, 3:240, or 5:240) were allocated to the experimental arenas. Adult♀ *X*. *flavipes* were selected from *X*. *flavipes* pure cultures when they were 5 to 8 days old and were assigned to experimental arenas. The selection of adult♀ *X. flavipes* of this age was considered because the time between adult♀ *X. flavipes* emergence and first oviposition is a minimum of four days. Two hundred and forty (240) adult♀ *L. decolor* were transferred into each experimental arena, with either 0, 1, 2, 3, or 5 adult♀ *X*. *flavipes*. The Control P-P ratio of 0:240 (no predators) is hereafter referred to as the Control P-P ratio. Adult♀ *X*. *flavipes* selected were starved for 24 h before introducing them to their prey to create a uniform hunger level, start a nomadic period, and minimize initial variability in oviposition [2,29]. The experimental arenas containing predators, and arenas without predators, were arranged randomly in plastic boxes with either NaNO_2_, NaCl, or KCl to maintain 63, 75, or 85% RH, respectively, as described in [18]. The RH boxes were subsequently maintained in growth chambers at temperatures of 20, 24, 28, or 32 °C for 40 days. The experimental design was a split–split plot in a randomized complete block design. The treatment structure was a 5 × 4 × 3 factorial. Factors were P-P ratios with five levels (0:240, 1:240, 2:240, 3:240, or 5:240), four levels of temperature (20, 24, 28, and 32 °C), and three levels of RH (63, 75, and 85%). There were a total of 60 factor level combinations (treatments), and each factor level combination was replicated four times except for the Control P-P ratio cases which had six replications. Experimental arenas were placed in the respective RH boxes and maintained at each of the four incubators. Each incubator (temperature level) contained all the combined levels of P-P ratio and RH, and all the treatment replications were run simultaneously. The number of nymphs and adults of *L*. *decolor* surviving were counted to estimate prey suppression by *X*. *flavipes* under the experimental conditions of different P-P ratios, temperatures, and RH after 40 days. Treatments with predators (1, 2, 3, or 5 predators) were compared with those without the predator (Control P-P ratio) for the different temperatures and RH conditions. For predator progeny production after 40 days, mobile stages (nymphs and adults) of *X*. *flavipes* under four P-P ratios with predators (1:240, 2:240, 3:240, or 5:240) and all temperature and RH combinations were counted and assessed.

### 2.5. Statistical Analysis

Generalized linear mixed-model methods were used to compare the number of *L. decolor* surviving after 40 days of exposure to *X*. *flavipes,* and the number of progeny produced by *X*. *flavipes* across the five P-P ratios (0:240, 1:240, 2:240, 3:240, or 5:240), four temperatures (20, 24, 28, and 32 °C), and three RH (63, 75, and 85%). PROC GLIMMIX in SAS models the main effects of the P-P ratio, temperature, and RH and their interactions for each of the response variables (number of *L. decolor* surviving and number of *X*. *flavipes* progeny produced) with the specified response distribution (~Poisson). For analyses involving percentage data, the beta distribution was specified in PROC GLIMMIX in SAS. Least squares means for appropriate significant effects were compared using the Tukey method. All data were analyzed using SAS software version 9.4 (SAS Institute, Cary, NC, USA), and tests were conducted at the nominal 0.05 level of significance.

## 3. Results

### 3.1. Effects of P-P Ratio, Temperature, and Relative Humidity on Survival of Liposcelis decolor

**Figure 1:**
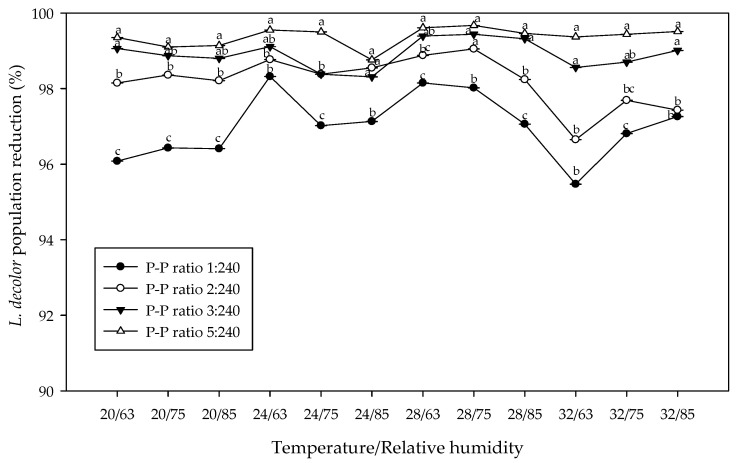
Percentage reduction in *Liposcelis decolor* population relative to Control predator–prey (P-P) ratio 0:240 when exposed to *Xylocoris flavipes* at four P-P ratios (1:240, 2:240, 3:240, and 5:240) under four levels of temperature (20, 24, 28, and 32° C) and three levels of relative humidity (RH) (63, 75, and 85%) over 40 days. Significant differences among P-P ratios for each temperature by relative humidity combination are denoted with different letters (*p* < 0.05, SAS, Tukey’s Honestly Significant Difference Test).

The results of the tests showed that the three-way interaction of P-P ratio, temperature, and RH with regard to *L. decolor* survival after exposure to *X. flavipes* for 40 days was significant (*p* < 0.05) (Table 1). Prey survival in the Control P-P ratio (0:240) was significantly higher compared to ratios with predators (1:240, 2:240, 3:240, and 5:240) in all the temperature and RH values (Table 2). Among ratios with predators, the lowest P-P ratio of 1:240 recorded the highest prey survival across all the different temperatures and RHs (Table 2). Compared with the Control P-P ratio, *X. flavipes* considerably suppressed *L. decolor* populations by ~99.47%, 99.06%, 98.25%, and 97.25% in the 5:240, 3:240, 2:240, and 1:240 P-P ratios, respectively, for the various temperature and RH combinations (Table 2 and Table 3, Figure 1). Generally, the highest number of surviving prey was found at a temperature of 32° C and 75% RH, which represent the optimal environmental conditions for *L. decolor* growth, development, and reproduction. At optimal conditions of 32 °C and 75% RH, the number of prey surviving in the Control P-P ratio was 3985.13 ± 255.45, compared with the range of 19.85 ± 2.47−115.73 ± 8.99 found for the four release ratios with the predator, representing a prey reduction of 97.10–99.50% (Table 2 and Figure 1). 

### 3.2. Effect of P-P Ratio, Temperature, and Relative Humidity on Xylocoris flavipes Progeny Production

There was no significant (*p* > 0.05) three-way interaction for P-P ratio, temperature, and RH in relation to *X*. *flavipes* progeny production after 40 days (Table 1). However, there was a significant interaction between temperature and P-P ratio (*p* < 0.05). Generally, more predator progeny were produced at P-P ratios of 3:240 and 5:240, especially at lower temperatures (20 and 24 °C) (Table 4). However, at higher temperatures (28 and 32 °C), which is more favorable for the prey, more progeny were produced at the 1:240 P-P ratio (Table 4). Relative humidity had no significant influence on *X*. *flavipes* progeny production (Table 1). Pooled analysis across relative humidity levels showed a significant temperature by P-P ratio interaction, and optimal *X*. *flavipes* progeny production occurred at the 1:240 P-P ratio at 28 and 32 °C (10.83 ± 0.95 and 8.50 ± 0.84, respectively) over 40 days (Table 5 and Table 6).

## 4. Discussion

Physical conditions in storage environments influence trophic-level interactions between predators and their prey and can affect the effectiveness of biocontrol agents [15,16]. Psocids are prolific, and their populations increase exponentially under favorable storage conditions of high temperatures and RHs [23,24]. Their high reproductive capacity, combined with their tolerance and resistance to phosphine and other insecticides, makes psocids difficult to control in storage facilities worldwide [23]. The results of the study showed that the presence of *X. flavipes* exerted high predation pressure on *L*. *decolor*, achieving prey suppression levels exceeding 97% across all the environmental conditions and P-P ratios compared with the Control ratio populations. The study suggests that *X*. *flavipes* can effectively manage psocids at temperatures and RHs typically found in most storage facilities. *Xylocoris flavipes* is a known predator of stored-product moths, beetles, and psocids, and it is registered in the United States by the Environmental Protection Agency (EPA) for use against stored-product insect pests [8,30,31,32]. A similar study by Danso et al. [28] found that two predatory mites, *C. eruditus* and *C. malaccensis* can effectively prey on *L. decolor* and suppress its population by 61.7–96.5% in 1:20, 2:20, 4:20, and 10:20 P-P ratios.

The commercialization and use of biocontrol agents to manage insect pests and mites in commodity storage systems, including seed stores, bulk-stored grain, bakeries, empty stores, and food processing companies, have been reported in Europe [6,33,34]. For example, in a bakery in Germany, the number of *Ephestia kuehniella* Zeller (Lepidoptera: Pyralidae) caught in pheromone traps decreased significantly over time when *Trichogramma evanescens* Westwood (Hymenoptera: Trichogrammatidae) was released at a rate of 25,000/week and *Habrobracon hebetor* (Say) (Hymenoptera: Braconidae) at a rate of 100/month [6]. Therefore, *X*. *flavipes* can be released inundatively to disinfest pallets, transportation containers, empty storehouses or warehouses, and storehouses with bagged commodities infested by psocids, potentially reducing the reliance on chemical pesticides.

The current study showed that *X*. *flavipes* caused >97% prey suppression at the optimal environmental conditions for *L. decolor* across all predator release ratios compared with the Control P-P ratio. The high prey population suppression achieved under favorable prey conditions demonstrates that *X. flavipes* has the ability to functionally respond to *L*. *decolor* population growth even at low P-P release ratios. A previous study by Bosomtwe et al. [8] indicated that nymphs and adults of *X. flavipes* display a Type II functional response when more *L*. *decolor* are available. The Type II functional response is characterized by an asymptotic relationship between predation rate and prey density due to handling time constraints [8,35]. The high prey suppression across all predator release ratios indicates *X*. *flavipes* maintain high per capita predation rates resulting in substantial population suppression even at the lowest P-P release ratio.

One of the findings from the current study is that higher release ratios result in greater prey population suppression compared to lower release ratios. This predator density-dependent suppression is consistent with findings from previous studies [2,18]. For example, a similar study by Danso et al. [18] showed that *L. decolor* population was significantly suppressed by *C. eruditus* by 61.7–87.5% when P-P release ratios were 1:20, 2:20, and 4:20, whereas the suppression levels of ~70.0–96.5% were observed for *C. malaccensis* at the same P-P release ratios in comparison with the populations in the Control ratio. A related study by Kucerova [2] found that at 1:2 P-P release ratio, the suppression rate of *L*. *decolor* population by *C. eruditus* was ~80.0% whereas at 1:5 release ratio, the suppression rate was ~84.2% compared with the Control ratio. Establishing a release ratio prior to the use of biocontrol agents is key to the success of biological control programs [27]. The predator density-dependent suppression observed in this study suggests that accurate estimation of release ratios, which ensure greater prey population suppression, is required. For instance, *Xylocoris flavipes* are cannibalistic, and individuals consume conspecifics under conditions of high predator density or limited prey [12]. This may divert predation pressure away from the target pests and reduce the overall efficacy of *X*. *flavipes*. Therefore, moderate release ratios would minimize the underutilization of the control potential of *X*. *flavipes* due to negative feedback factors including cannibalism, competition, and mutual interference [18,36]. Moreover, this information is important for mass rearing and commercial production of *X. flavipes* where release of a lower number of predators is recommended.

Progeny production of *X. flavipes* revealed interaction between temperature and P-P release ratio in which at higher temperatures, more progeny were produced at the lowest P-P release ratio, whereas at lower temperatures, *X*. *flavipes* produced more progeny at the highest P-P release ratio. This observation contrasts the study by Danso et al. [18], who found that *C. malaccensis* and *C*. *eruditus* increased their progeny production at decreasing P-P release ratios and temperatures and increasing RH. The contradictory trend indicates that the different predator species use distinct ecological strategies to optimize reproduction. *Xylocoris flavipes* is cannibalistic, and increased predator metabolic activity at higher temperatures may have resulted in rapid depletion of prey resources, triggering conspecifics cannibalism at the highest release ratios [12]. However, the lowest release ratio maintained sufficient prey to sustain predator progeny production with minimal cannibalism at the higher temperatures. Although lower temperatures are known to be less favorable for the survival of *X. flavipes* [17], higher release ratios are likely to increase the probability of predator progeny survival. This may explain the increased number of progeny at the highest P-P ratio (5:240) compared with the lowest P-P ratio (1:240) at the lower temperatures. Therefore, for inoculative release of *X*. *flavipes* to manage psocid infestations, low to moderate release ratios should be targeted in warmer storage conditions (≥28 °C) to minimize cannibalism and ensure predator establishment. However, in cooler conditions (≤24 °C), higher release ratios would be required to compensate for reduced predator survival. Because RH was not found to be a limiting factor to predation and progeny production in this study, it suggests that *X*. *flavipes* can be released in a range of storage environment RH conditions without compromising effectiveness. In the case of *C*. *eruditus* and *C. malaccensis*, 63% RH was found to be detrimental to population growth and survival of both predators [18]. The tolerance of *X*. *flavipes* to varying moisture conditions compared to the two predatory mites represents a key practical advantage for *X*. *flavipes* deployment in storage facilities where maintaining optimal humidity for the predatory mites may not be feasible.

## 5. Conclusions

This study demonstrated that *X*. *flavipes* can effectively manage *L*. *decolor* populations and cause prey suppression rate of >97% across all P-P release ratios and environmental conditions tested. Maximum prey suppression rate of ~99.47% was achieved at the highest P-P release ratio (5:240). There were interaction effects between temperature and P-P release ratio on predator progeny production. Relative humidity had no detrimental effect on the performance of *Xylocoris flavipes*, suggesting application in a wide range of storage environmental conditions. For biocontrol applications, lower P-P release ratios (1:240 and 2:240) should be targeted in warmer storage conditions to minimize cannibalism and ensure predator establishment. Further evaluation with a wider range of release ratios under field conditions and assessment of long-term predator sustainability is required. Moreover, studies on the compatibility of *X*. *flavipes* with other predators such as *C*. *eruditus* and *C. malaccensis*, evaluation against other economically important *Liposcelis* species, including *L*. *bostrychophila*, *L*. *entomophila*, and *L*. *paeta*, and the impact of pesticides on *X*. *flavipes* survival should be investigated to facilitate integration into storage IPM systems for the management of psocids.

## Figures and Tables

**Table 1:** Summary of tests for main effects of predator–prey (P-P) ratio, temperature (T), and relative humidity (RH), and interactions for number of prey surviving (*Liposcelis decolor*) and predator progeny production of *Xylocoris flavipes* exposed to initial prey density of 240 females of *L*. *decolor* over 40 days.

Variable	Source	Df	*F*	*p*-Value
Prey survival	T	3, 15	57.42	<0.0001
	RH	2, 40	71.92	<0.0001
	T*RH	6, 40	5.77	0.0002
	P-P	4, 144	19904.00	<0.0001
	T*P-P	12, 144	32.00	<0.0001
	RH*P-P	8, 144	5.60	<0.0001
	T*RH*P-P	24, 144	11.14	<0.0001
Predator progeny	T	3, 9	9.58	0.0037
	RH	2, 24	2.51	0.1025
	T*RH	6, 24	1.82	0.1373
	P-P	3, 108	2.83	0.0419
	T*P-P	9, 108	3.63	0.0005
	RH*P-P	6, 108	0.31	0.9317
	T*RH*P-P	18, 108	0.47	0.9663

Asterisk (*) denotes interactions between variables predator–prey (P-P) ratio, temperature (T), and relative humidity (RH).

**Table 2:** Mean number of *Liposcelis decolor* surviving (±SE) over 40 days. Predator was *Xylocoris flavipes*; initial prey density was 240 females of *L. decolor*; there were five levels of predator–prey (P-P) ratio (0:240, 1:240, 2:240, 3:240, and 5:240), four levels of temperature (T) (20, 24, 28, and 32° C), and three levels of relative humidity (RH) (63, 75, and 85%).

Temperature (T)	Relative Humidity (RH)	Predator-Prey (P-P) Ratio				
		**0:240**	**1:240**	**2:240**	**3:240**	**5:240**
20	63	772.60 ± 50.56 aD	32.84 ± 3.60 bE	15.00 ± 2.19 cF	7.24 ± 1.45 dE	5.69 ± 1.27 dE
20	75	1134.33 ± 73.64 aC	40.43 ± 4.08 bD	18.38 ± 2.43 cE	12.01 ± 1.88 dD	9.31 ± 1.62 dE
20	85	1140.50 ± 74.03 aC	42.35 ± 4.26 bD	20.54 ± 2.64 cE	13.19 ± 2.02 dD	9.89 ± 1.71 dE
24	63	1850.06 ± 119.29 aB	31.48 ± 3.46 bE	22.41 ± 2.78 cE	15.86 ± 2.24 dD	7.30 ± 1.43 eE
24	75	2176.50 ± 140.10 aB	62.59 ± 5.56 bC	35.90 ± 3.66 cC	15.65 ± 2.17 dD	10.43 ± 1.71 dD
24	85	2028.08 ± 130.64 aB	60.93 ± 5.55 bC	29.70 ± 3.35 cD	37.38 ± 3.91 cB	24.83 ± 2.98 dA
28	63	1850.68 ± 119.33 aB	34.00 ± 3.65 bE	21.66 ± 2.72 cE	11.59 ± 1.86 dD	6.54 ± 1.35 eE
28	75	3340.82 ± 214.36 aA	61.60 ± 5.47 bC	29.17 ± 3.21 cD	16.80 ± 2.25 dD	7.93 ± 1.45 eE
28	85	3393.07 ± 217.69 aA	98.79 ± 8.01 bA	59.32 ± 5.38 cB	23.29 ± 2.82 dC	16.67 ± 2.29 eC
32	63	1282.16 ± 83.07 aC	58.49 ± 5.31 bC	44.77 ± 4.37 cC	19.50 ± 2.50 dC	7.70 ± 1.45 eE
32	75	3985.13 ± 255.45 aA	115.73 ± 8.99 bA	82.79 ± 6.83 cA	45.57 ± 4.33 dA	19.85 ± 2.47 eB
32	85	3512.91 ± 225.33 aA	88.54 ± 7.25 bB	82.53 ± 6.85 bA	31.44 ± 3.36 cB	14.10 ± 2.01 dD

Significant differences among P-P ratios for each T*RH combination are denoted with different lowercase letters (within the same row) and differences among T*RH combinations for each P-P ratio are denoted by different uppercase letters (within column), (*p* < 0.05, SAS, Tukey’s Honestly Significant Difference Test).

**Table 3:** Summary of tests for main effects of predator–prey (P-P) ratio, temperature (T), and relative humidity (RH), and interactions for percentage reduction in *Liposcelis decolor* population relative to Control P-P ratio 0:240 when exposed to *Xylocoris flavipes* for 40 days.

Variable	Source	Df	*F*	*p*-Value
Prey population reduction (%)	T	3, 144	17.60	<0.0001
	RH	2, 144	3.55	<0.0312
	T*RH	6, 144	5.25	<0.0001
	P-P	3, 144	160.42	<0.0001
	T*P-P	9, 144	4.24	<0.0001
	RH*P-P	6, 144	0.75	0.6075
	T*RH*P-P	18, 144	11.14	0.8408

Asterisk (*) denotes interactions between variables predator–prey (P-P) ratio, temperature (T), and relative humidity (RH).

**Table 4:** Mean number of progeny (±SE) of *Xylocoris flavipes* over 40 days. Initial prey density was 240 females of *Liposcelis decolor*, four levels of predator–prey (P-P) ratio (1:240, 2:240, 3:240, and 5:240), four levels of temperature (T) (20, 24, 28, and 32 °C), and three levels of relative humidity (RH) (63, 75, and 85%).

Temperature (T)	Relative Humidity (RH)	Predator–Prey (P-P) Ratio			
		**1:240**	**2:240**	**3:240**	**5:240**
20	63	3.50 ± 0.94 Db	5.75 ± 1.20 aB	5.25 ± 1.15 aB	7.00 ± 1.32 a
20	75	4.25 ± 1.03 bD	5.75 ± 1.20 aB	6.50 ± 1.27 aAB	7.75 ± 1.39 a
20	85	3.75 ± 0.97 bD	3.50 ± 0.93 bC	5.00 ± 1.12 aB	7.00 ± 1.32 a
24	63	6.50 ± 1.27 bBC	7.50 ± 1.37 bAB	8.50 ± 1.46 abA	9.50 ± 1.54 a
24	75	3.50 ± 0.93 bD	5.00 ± 1.12 aB	6.25 ± 1.25 aAB	7.75 ± 1.39 a
24	85	5.25 ± 1.15 aC	4.75 ± 1.09 aB	5.50 ± 1.17 aB	6.50 ± 1.27 a
28	63	13.50 ± 1.83 aA	7.75 ± 1.39 bAB	7.25 ± 1.35b AB	8.00 ± 1.41 b
28	75	10.00 ± 1.58 aB	8.00 ± 1.41 aAB	6.00 ± 1.22 aAB	7.25 ± 1.35 a
28	85	9.00 ± 1.50 aB	6.50 ± 1.27 abAB	5.75 ± 1.20 bB	7.50 ± 1.37 ab
32	63	8.75 ± 1.48 aB	6.00 ± 1.22 bAB	5.50 ± 1.17 bB	8.00 ± 1.41 a
32	75	9.00 ± 1.50 aB	9.00 ± 1.50 aA	5.75 ± 1.20 bB	7.25 ± 1.35 ab
32	85	7.75 ± 1.39 aBC	7.50 ± 1.37 aAB	7.75 ± 1.39 aAB	7.00 ± 1.32 a

Significant differences among P-P ratios for each T*RH combination are denoted with different lowercase letters (within the same row) and differences among T*RH combinations for each P-P ratio are denoted by different uppercase letters (within column), (*p* < 0.05, SAS, Tukey’s Honestly Significant Difference Test).

**Table 5:** Summary of tests for main effects of predator–prey (P-P) ratio, temperature (T), and interactions for number of *Xylocoris flavipes* progeny production when exposed to initial prey density of 240 females of *L*. *decolor* over 40 days. Data was pooled across three levels of relative humidity based on non-significant RH effects (*p* > 0.05) (Table 1).

Variable	Source	Df	*F*	*p*-Value
Predator progeny	T	3, 176	11.79	<0.0001
	P-P	3, 176	3.62	0.0143
	T*P-P	9, 176	5.09	<0.0001

**Table 6:** Mean number of progeny (±SE) of *Xylocoris flavipes* under four temperature levels and four predator–prey (P-P) ratios, with data pooled across three levels of relative humidity based on non-significant RH effects. Initial prey density was 240 females of *Liposcelis decolor*.

Temperature (T)	Predator-Prey (P-P) Ratio			
	**1:240**	**2:240**	**3:240**	**5:240**
20	3.25 ± 0.52 bB	5.00 ± 0.65 abA	5.58 ± 0.68 abA	7.25 ± 0.78 aA
24	4.08 ± 0.58 bB	5.75 ± 0.69 abA	6.75 ± 0.75 aA	7.92 ± 0.81 aA
28	10.83 ± 0.95 aA	7.42 ± 0.79 abA	6.33 ± 0.73 bA	7.58 ± 0.79 abA
32	8.50 ± 0.84 aA	7.50 ± 0.79 aA	6.33 ± 0.72 aA	7.42 ± 0.79 aA

Significant differences among P-P ratios for each temperature (T) are denoted with different lowercase letters (within the same row) and differences among temperatures (T) for each P-P ratio are denoted by different uppercase letters (within column), (*p* < 0.05, SAS, Tukey’s Honestly Significant Difference Test).

## Data Availability

The original contributions presented in this study are included in the article. Further inquiries can be directed to the corresponding author.
